# Latitudinal Diversity of *Culex pipiens* Biotypes and Hybrids in Farm, Peri-Urban, and Wetland Habitats in Europe

**DOI:** 10.1371/journal.pone.0166959

**Published:** 2016-11-21

**Authors:** Chantal B. F. Vogels, Tim W. R. Möhlmann, Diede Melsen, Guido Favia, Uno Wennergren, Constantianus J. M. Koenraadt

**Affiliations:** 1 Laboratory of Entomology, Wageningen University and Research centre, Wageningen, The Netherlands; 2 IFM Theory and Modelling, Linköping University, Linköping, Sweden; 3 School of Biosciences and Veterinary Medicine, University of Camerino, Camerino, Italy; Universidade Nova de Lisboa Instituto de Higiene e Medicina Tropical, PORTUGAL

## Abstract

Despite the presence of *Culex (Cx*.*) pipiens* mosquitoes and circulation of West Nile virus (WNV), WNV outbreaks have so far not occurred in northern Europe. The species *Cx*. *pipiens* consists of two morphologically identical biotypes, *pipiens* and *molestus*, which can form hybrids. Until now, population dynamic studies of *Cx*. *pipiens* have not differentiated between biotypes and hybrids at the European scale, nor have they used comparative surveillance approaches. We therefore aimed to elucidate the relative abundance of *Cx*. *pipiens* biotypes and hybrids in three habitat types at different latitudes across Europe, using two different surveillance traps. BG-Sentinel and Mosquito-Magnet Liberty Plus traps were placed in three habitat types (farms, peri-urban, wetlands), in three European countries (Sweden, The Netherlands, Italy). Collected *Cx*. *pipiens* mosquitoes were identified to biotype with real-time PCR. Both trap types collected equal ratios of the biotypes and their hybrids. From northern to southern latitudes there was a significant decrease of *pipiens* and an increase of *molestus*. Habitat types influenced the relative ratios of biotypes and hybrids, but results were not consistent across latitudes. Our results emphasize the need to differentiate *Cx*. *pipiens* to the biotype level, especially for proper future WNV risk assessments for Europe.

## Introduction

Global warming, increased travel and trade, and land-use changes are important drivers for the (re-)emergence of vector-borne diseases, such as West Nile virus (WNV; family: *Flaviviridae*) [1]. The potential of WNV to quickly spread to new areas is clearly illustrated by the outbreaks that occurred in the United States of America, after the initial introduction in 1999 [2–4]. WNV outbreaks have also occurred in southern and central European countries, but no outbreaks among humans have occurred in northern Europe [5–7].

WNV is maintained in an enzootic cycle between birds and mosquitoes. The main vector for WNV is the mosquito *Culex (Cx*.*) pipiens* [8,9]. The *Cx*. *pipiens* complex consists of several closely related species and biotypes, of which only the species *Cx*. *pipiens* (Linnaeus 1758) occurs in Europe [10]. Because of its similar morphology, the species *Cx*. *torrentium* (Martini 1925) is often included in taxonomic studies of the *Cx*. *pipiens* complex [11]. The species *Cx*. *pipiens* consists of two morphologically similar biotypes, named *pipiens* (Linnaeus 1758) and *molestus* (Forskål 1775), which show distinct behaviour. Biotype *pipiens* is the most important vector in the enzootic cycle because of its preference for birds [12]. During winter, biotype *pipiens* enters diapause, which provides a means of overwintering for WNV [13,14]. Biotype *molestus* prefers mammals, including humans, as hosts, and remains active year-round [15–17]. Host availability can induce a strong shift in host feeding behaviour of biotype *molestus* from mammals to birds, especially in areas with high bird densities [18]. Previously, biotype *molestus* has been described as occurring underground [8], but recent studies show that both biotypes occur sympatrically in aboveground habitats throughout Europe [19–22]. Furthermore, biotype *pipiens* and biotype *molestus* can form hybrids which show intermediate host preference [23]. As a result of this, hybrids can play an important role in bridging WNV from birds to humans [8].

Several studies elucidated the geographic distribution of the species *Cx*. *pipiens* and *Cx*. *torrentium* (Martini 1925) at the European scale [24,25]. In general, *Cx*. *torrentium* is relatively more abundant in northern Europe, whereas *Cx*. *pipiens* is more abundant in southern Europe [24]. However, these studies did not identify *Cx*. *pipiens* mosquitoes to the biotype level. Identification to the biotype level is important because the behavioural differences between the two biotypes of *Cx*. *pipiens* and their hybrids result in different vectorial capacity for WNV. Thus far, in-depth studies that differentiated between the biotypes were done at country level [19–22,26,27]. Few of these studies systematically compared biotype ratios among different habitat types [19,20,27]. However, due to differences in experimental design it is hard to make direct comparisons between *Cx*. *pipiens* populations in northern and southern European countries.

The aim of this study was to assess the relative abundance of the *Cx*. *pipiens* biotypes with two types of traps (Biogents Sentinel and Mosquito Magnet Liberty Plus), in three different habitat types (farms, peri-urban, and wetlands), and in three countries (Sweden, The Netherlands, and Italy) at different latitudes across Europe.

## Materials & Methods

### Ethics statement

Permits and approval for field work in wetlands were obtained from the county board of Östergötland in Sweden, Staatsbosbeheer in The Netherlands, and the Protected Areas Service of the San Benedetto del Tronto Municipality in Italy. For farms and peri-urban habitats approval was obtained from landowners of private properties in all three countries. No protected species were sampled in this study.

### Mosquito collections

Adult mosquitoes were collected with the Biogents Sentinel (BGS) trap (BioGents GmbH, Germany) and the Mosquito Magnet Liberty Plus (MMLP) trap (Woodstream Corp., USA). A mixture of 17.5 g dry instant yeast (Bruggeman, The Netherlands), 250 g white granulated sugar and 2 l of tap water in a 5 l plastic bottle was used for CO_2_ production in the BGS trap [28]. Combustion of propane provided CO_2_ for the MMLP trap.

Both traps were rotated among three trapping locations, in three different habitat types (farms, peri-urban, and wetlands), in Sweden (Linköping), The Netherlands (Wageningen), and Italy (San Benedetto del Tronto; Table 1). The selected farms were dairy cattle farms with a minimum of 10 cows. Traps were placed within 50 m of the open indoor stable. Peri-urban locations were at the periphery of a city (inhabitants <150,000), and within a 50 m radius of the trap, at least two occupied residential properties were present. Locations in a wetland habitat had a minimum of 50% marshy or standing water within a 100 m radius of the traps. Trapping locations were at least 100 m apart.

**Table 1:** Coordinates of all 27 trapping locations in the three different habitat types (farms, peri-urban, and wetlands) in three different European countries (Sweden, The Netherlands, and Italy).

Country	Habitat type	Sampling location	Coordinates
Sweden	Farms	1	58.296530, 15.584782
(Linköping)		2	58.343622, 15.602404
		3	58.330597, 15.704327
	Peri-urban	4	58.416973, 15.499516
		5	58.401515, 15.626744
		6	58.405494, 15.595035
	Wetlands	7	58.362106, 15.651861
		8	58.361585, 15.654910
		9	58.361542, 15.659072
The Netherlands	Farms	10	51.971084, 5.761455
(Wageningen)		11	51.973637, 5.773978
		12	52.013077, 5.645998
	Peri-urban	13	52.018075, 5.655372
		14	51.979257, 5.645230
		15	51.979771, 5.660278
	Wetlands	16	51.969443, 5.758940
		17	51.967693, 5.758896
		18	51.971671, 5.747826
Italy	Farms	19	42.914466, 13.854588
(San Benedetto del Tronto)		20	42.944809, 13.859857
		21	42.943098, 13.853856
	Peri-urban	22	42.883455, 13.879388
		23	42.951012, 13.850783
		24	42.934424, 13.891933
	Wetlands	25	42.896600, 13.911895
		26	42.899042, 13.909813
		27	42.903365, 13.908667

Collections were done during six consecutive days, every month in each country. Sampling periods were from July 2014 to June 2015, except for the winter months December, January, and February (and March for Sweden). Traps were emptied and repositioned every 24 hours between sunrise and sunset of the next day. Mosquitoes were stored at -20°C in Eppendorf tubes containing small silica beads covered with cotton wool.

### Mosquito identifications

All female mosquitoes were identified to species level, following the European identification key for female mosquitoes [29]. The number of *Cx*. *pipiens* mosquitoes captured each month was not sufficient to statistically test for temporal differences in biotype and hybrid ratios. Therefore, all 190 *Cx*. *pipiens* females available for analysis from Sweden, and a selection of 300 *Cx*. *pipiens* females from Italy and 299 *Cx*. *pipiens* females from The Netherlands were used, resulting in a total of 789 mosquitoes analysed. Samples from the Netherlands and Italy were partially random selected with 100 samples per habitat, for both countries.

Selected mosquitoes were further identified to species (*Cx*. *pipiens* or *Cx*. *torrentium*) and biotype (*pipiens*, *molestus*, or hybrid) level. We followed the real-time PCR assay for differentiation between the *Cx*. *pipiens* biotypes as described in detail before [22]. Briefly, for *Cx*. *pipiens* we used forward and reverse primers Cx_pip_F (5’-GCGGCCAAATATTGAGACTTTC-3’) and Cx_pip_R (5’-ACTCGTCCTCAAACATCCAGACATA-3’). For identification of biotype *molestus* we used probe Cpp_mol_P (5’-FAM-TGAACCCTCCAGTAAGGTA-MGB-3’), and for biotype *pipiens* we used the two probes Cpp_pip_P1 (5’-VIC-CACACAAAYCTTCACCGAA-MGB-3’) and Cpp_pip_P2 (5’-VIC-ACACAAACCTTCATCGAA-MGB-3’). Hybrids were identified when both probes for biotype *pipiens* and *molestus* were amplified by real-time PCR. For identifications of *Cx*. *torrentium* we used forward and reverse primers Cx_tor_F (5’-CTTATTAGTATGACACAGGACGACAGAAA-3’) and Cx_tor_R (5’-GCATAAACGCCTACGCAACTACTAA-3’), and probe Cx_tor_P (5’-FAM-ATGATGCCTGTGCTACCA-MGB-3’). Thermocycler conditions were 95°C for 10 min, followed by 45 cycles of 95°C for 15 s and 62°C for 1 min. The PCR was run on the CFX96 Real-Time PCR system (Bio-Rad Laboratories, Hercules, CA) and data were analysed in CFX manager 2.0 (Bio-Rad Laboratories, Hercules, CA).

### Statistical analyses

Main effects (trap type, country, and habitat) and within-effects (habitats within each country, and country within each habitat) on the ratios of *Cx*. *pipiens* mosquitoes were tested with Pearson’s Chi-square tests. Significant effects were further evaluated with pairwise comparisons and corrected with the Bonferroni correction. All data were analysed in the statistical software package R [30].

## Results

In total 5,202 *Cx*. *pipiens* females were collected of which 3,878 females were collected with the BGS trap and 1,324 females with the MMLP trap (Table 2). Of the 789 mosquitoes selected for analysis, 663 mosquitoes were identified as *Cx*. *pipiens*, of which 463 (69.8%) were identified as biotype *pipiens*, 127 (19.2%) as biotype *molestus*, and 73 (11.0%) as hybrids (S1 Dataset). In addition, 14 mosquitoes were identified as *Cx*. *torrentium*, which all originated from Sweden. The number of *Cx*. *torrentium* mosquitoes was too low for reliable statistical tests, and these samples were therefore excluded from further analyses. The remaining 112 mosquitoes did not amplify a PCR product.

**Table 2:** Total number of collected *Cx*. *pipiens* females per trap type, habitat, and country. BGS = Biogents Sentinel trap, MMLP = Mosquito Magnet Liberty Plus trap, SW = Sweden, NL = The Netherlands, and IT = Italy.

	BGS			MMLP		
	SW	NL	IT	SW	NL	IT
Farms	19	252	128	29	64	21
Peri-Urban	56	1063	111	44	451	37
Wetlands	33	969	1247	24	101	553
Total	108	2284	1486	97	616	611

**Fig 1:**
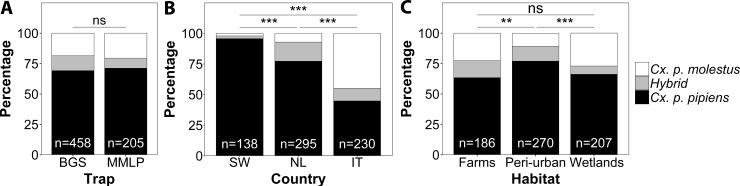
**Main effects of (A) trap type, (B) country, and (C) habitat on the ratio of *Culex pipiens* biotypes and their hybrids.** The total sample size (n) is indicated for each bar. Significance is displayed for each pairwise comparison, with ns = not significant, ** = p < 0.01, *** = p < 0.001. BGS = Biogents Sentinel trap, MMLP = Mosquito Magnet Liberty Plus, SW = Sweden, NL = The Netherlands, and IT = Italy.

Both trap types, BGS and MMLP, trapped similar ratios of the *Cx*. *pipiens* biotypes and hybrids (χ^2^ = 2.35, df = 2, p = 0.31; Fig 1A). Thus, data from both trap types were pooled for further analyses.

The ratios of *Cx*. *pipiens* biotypes and hybrids were significantly different between the three countries in Europe (χ^2^ = 173.62, df = 4, p<0.001; Fig 1B). Pairwise comparisons between countries showed that *Cx*. *pipiens* ratios were different between each combination of Italy, The Netherlands, and Sweden (all pairwise comparisons: p<0.001). The proportion of biotype *pipiens* was highest in Sweden (90%) and gradually decreased towards more southern latitudes, with the lowest proportion of biotype *pipiens* in Italy (40%).

The ratios of *Cx*. *pipiens* biotypes and hybrids were also significantly different between habitat types (χ^2^ = 26.59, df = 4, p<0.001; Fig 1C). Peri-urban habitats had a relatively higher proportion of biotype *pipiens* compared to both farms (p<0.01), and wetlands (p<0.001). There was no difference in ratios between farms and wetlands (p = 0.16).

**Fig 2:**
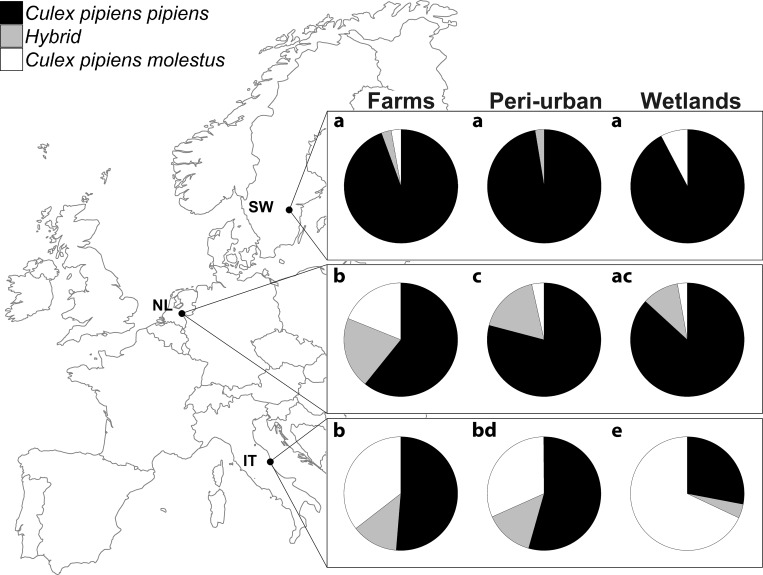
Within-effect of habitat within each of the three countries on the ratio of *Culex pipiens* biotypes and their hybrids (rows), and within-effect of country within each habitat type (columns). The sample size for each pie chart ranges between n = 26–115. Letters display significant differences between ratios shown in rows and columns, at a significance level of p < 0.05. SW = Sweden, NL = The Netherlands, and IT = Italy.

In order to gain more insight in the interaction between country and habitat, pairwise comparisons were made between the habitats within each country, and the countries within each habitat type (Fig 2). Ratios of *Cx*. *pipiens* biotypes and hybrids were significantly different between habitats in Italy (χ^2^ = 25.05, df = 4, p<0.001) and The Netherlands (χ^2^ = 26.37, df = 4, p<0.001), but were similar within Sweden (χ^2^ = 6.11, df = 4, p = 0.19; Fig 2). In The Netherlands, farms were different due to the relatively high proportion of biotype *molestus* and hybrids (p<0.01), whereas in Italy wetlands were different due to the high proportion of biotype *molestus* (p<0.001).

Ratios of *Cx*. *pipiens* biotypes and hybrids were significantly different between countries within each of the habitat types (p<0.001; Fig 2). Farms in Sweden had a relatively higher proportion of biotype *pipiens* compared to Italy (p<0.001) and The Netherlands (p<0.01), which both had relatively more biotype *molestus* and hybrids. For peri-urban habitats, ratios were significantly different among all countries (pairwise comparisons: p≤0.001), with a gradual increase of biotype *pipiens* towards northern latitudes. Wetlands in Italy had a relatively low proportion of biotype *pipiens* but high proportion of biotype *molestus*, compared to The Netherlands (p<0.001) and Sweden (p<0.001), which both had relatively high proportions of biotype *pipiens*.

## Discussion

The aim of this study was to assess the relative abundance of the *Cx*. *pipiens* biotypes and their hybrids in different habitats from northern to southern latitudes in Europe, using two trap types. We found a strong latitudinal effect on the ratios of the *Cx*. *pipiens* biotypes and hybrids, with a gradient of decreasing biotype *pipiens* from northern to southern latitudes. Habitat types also influenced the ratios of *Cx*. *pipiens* biotypes and hybrids, but effects were not consistent at the different latitudes.

Due to low numbers, *Cx*. *torrentium* was excluded from the analyses in this study. All *Cx*. *torrentium* females that we identified originated from Sweden, whereas no *Cx*. *torrentium* females were found in The Netherlands and Italy. These results are consistent with previous studies that showed a relatively high abundance of *Cx*. *torrentium* in northern European countries [24,31], and relatively low abundance or even absence in southern Europe [19].

No difference was found between the ratio of *Cx*. *pipiens* biotypes and their hybrids collected with the BGS and MMLP traps. Despite the differences in trapping mechanism between the two traps, there was no apparent difference in the attraction of the biotypes and their hybrids towards both traps. For studies focusing on relative abundance both traps can thus be used equally well, but differences in the total numbers of collected *Cx*. *pipiens* mosquitoes between both traps do exist. Results of studies on mosquito abundance with different traps may not be directly comparable.

Our study shows the sympatric occurrence of both *Cx*. *pipiens* biotypes and hybrids in aboveground habitats throughout Europe. These results are in line with previous findings from The Netherlands [22], Germany [21], Austria [27], Portugal [20,32], Italy [19], and Greece [26]. There was a clear gradient of decreasing biotype *pipiens* and increasing biotype *molestus* proportions from northern to southern latitudes. In Sweden the major part of the *Cx*. *pipiens* mosquitoes collected consisted of the *pipiens* biotype, whereas ratios in Italy were more equal between *pipiens* and *molestus* biotypes. This pattern was visible when ratios were determined per country without differentiating between the different habitat types, as well as when ratios for each country were split over the three habitat types. Previously, only a single record of the *molestus* biotype was known for Sweden [33]. In addition, the proportion of hybrids in Sweden was much lower compared to The Netherlands and Italy, which can be explained by the near absence of biotype *molestus* in all habitat types in Sweden. In this study we confirm that both biotypes and their hybrids occur aboveground at latitudes up to 58°24'36"N.

All peri-urban habitats combined had a relatively higher proportion of biotype *pipiens* and fewer biotype *molestus* compared to farms and wetlands. This pattern was, however, not consistent when comparing habitats within each of the three countries. In Sweden the ratios were similar for the three habitats, whereas in The Netherlands farms and in Italy wetlands ratios were different from the other two habitat types. This inconsistency could be explained by differences in, for instance, climate, microhabitat, availability of breeding sites, and hosts which all may influence the presence of the biotypes. These factors are likely to differ more between countries at different latitudes than between nearby habitats at one geographic location. Our findings also show variation at the local scale between habitats in The Netherlands and Italy. Especially the Italian wetlands stand out because of the high proportion of biotype *molestus*. Although a higher proportion of biotype *pipiens* was expected in such bird-rich habitats, a previous study showed high proportions of biotype *molestus* up to 82% in both urban and rural habitats with aboveground breeding sites in Italy [19]. The same study also showed high variation in *Cx*. *pipiens* biotype composition throughout Italy [19]. The ratios that we found for each country do, therefore, not represent an overall ratio of *Cx*. *pipiens* for the entire country, but rather for the specific sampling location. The sampling strategy used in our study is suitable for direct comparisons between locations at different latitudes, due to the consistent design over all countries. Studies that place traps at random locations throughout a country are more useful to get insight in local variation and dynamics of *Cx*. *pipiens* within a country [19–22,27].

*Cx*. *pipiens* populations that are dominated by biotype *pipiens* play an important role in the natural transmission cycle of WNV in birds, whereas the risk of WNV outbreaks among humans is increased in populations with high levels of hybridization [8]. Up to now, outbreaks of WNV among humans have only occurred in southern and central Europe, including Italy [34]. The overall proportion of hybrids is higher in The Netherlands than Italy, which is not consistent with the more equal proportion of biotypes in Italy than in The Netherlands. This indicates a more complex cause of hybridization than solely density dependence. If WNV would get established in The Netherlands, the higher degree of hybridization may result in a higher likelihood of bridging of WNV from birds to humans. However, other factors such as vector competence and climate determine whether transmission cycles can get established. Such factors are most likely limiting the transmission of WNV in northern Europe [35,36]. Future studies on vector competence of the *Cx*. *pipiens* biotypes and hybrids under different climatic scenarios are needed in order to gain more insight in the risk of the transmission of viruses by mosquitoes in Europe.

## Conclusions

The BGS and MMLP traps collected equal ratios of the *Cx*. *pipiens* biotypes and their hybrids. A clear gradient of decreasing biotype *pipiens* and increasing biotype *molestus* proportions from northern to southern latitudes in Europe was found. Hybrids were found in all countries, but highest proportions were recorded in The Netherlands and Italy. Furthermore, *Cx*. *pipiens* ratios between habitat types were different. These differences were, however, not consistent when comparing habitat types within countries. Future research should focus on, (i) vector competence of *Cx*. *pipiens* biotypes and hybrids at different latitudes in Europe to assess the risks of WNV transmission in northern Europe, and (ii) the ecology of hybrids in order to estimate the risk of WNV being transmitted to humans.

## Supporting Information

S1 Dataset Results of *Cx. pipiens* biotype analyses. (XLSX)
